# Influence of feeding practices in the composition and functionality of infant gut microbiota and its relationship with health

**DOI:** 10.1371/journal.pone.0294494

**Published:** 2024-01-03

**Authors:** Misael Martínez-Martínez, Marco Martínez-Martínez, Ruth Soria-Guerra, Sandra Gamiño-Gutiérrez, Carolina Senés-Guerrero, Arlette Santacruz, Rogelio Flores-Ramírez, Abel Salazar-Martínez, Diana Portales-Pérez, Horacio Bach, Fidel Martínez-Gutiérrez

**Affiliations:** 1 Facultad de Ciencias Quimicas, Universidad Autonoma de San Luis Potosí, SLP, Mexico; 2 Instituto Mexicano del Seguro Social, Torreón, Mexico; 3 Tecnológico de Monterrey, Campus Guadalajara, Zapopan, Jalisco, Mexico; 4 Posgrado en Biotecnología, Centro de Biotecnología FEMSA, Tecnológico de Monterrey, Monterrey, Mexico; 5 Laboratorio de Salud Total, Centro de Investigación Aplicada en Ambiente y Salud -CIACYT, Universidad Autónoma de San Luis Potosí, SLP, Mexico; 6 Hospital Central “Dr. Ignacio Morones Prieto”, San Luis Potosí, SLP, Mexico; 7 Centro de Investigación en Ciencias de la Salud y Biomedicina, Universidad Autonoma de San Luis Potosí, SLP, Mexico; 8 Department of Medicine, Division of Infectious Diseases, University of British Columbia, Vancouver, BC, Canada; Satyawati College, University of Delhi, INDIA

## Abstract

Establishing the infant’s gut microbiota has long-term implications on health and immunity. Breastfeeding is recognized as the best practice of infant nutrition in comparison with formula feeding. We evaluated the effects of the primary feeding practices by analyzing the infant growth and the potential association with gut diseases. A cross-sectional and observational study was designed. This study included 55 mothers with infants, who were divided according to their feeding practices in breastfeeding (BF), formula feeding (FF), and combined breast and formula feeding (CF). Anthropometric measurements of the participants were recorded. Additionally, non-invasive fecal samples from the infants were collected to analyze the microbiota by sequencing, immunoglobulin A (IgA) concentration (ELISA), and volatile organic compounds (gas chromatography with an electronic nose). Results showed that the microbiota diversity in the BF group was the highest compared to the other two groups. The IgA levels in the BF group were twice as high as those in the FF group. Moreover, the child´s growth in the BF group showed the best infant development when the data were compared at birth to the recollection time, as noted by the correlation with a decreased concentration of toxic volatile organic compounds. Interestingly, the CF group showed a significant difference in health status when the data were compared with the FF group. We conclude that early health practices influence children’s growth, which is relevant to further research about how those infants’ health evolved.

## Introduction

The human microbiota is composed mainly of bacteria but also includes methanogenic archaea, fungi, protozoa, and viruses that colonize the skin and the respiratory, genitourinary, and gastrointestinal tracts [1]. The development of the intestinal microbiota differs considerably between infants and depends upon multiple extrinsic factors, including mode of infant delivery, type of nutrition, use of antimicrobials [2], and environmental exposures [3]. The World Health Organization recognizes that breastfeeding (BF) is the optimal feeding process and recommends that all infants must be exclusively breastfed in the first 6 months of life [4]. Breast milk provides a complex community of microorganisms that will colonize the digestive tract [5] and human milk oligosaccharides (HMO), both with a close relationship to the infant gut microbiota [6]. Various authors have shown evidence that children with BF were protected against child infection, increased intelligence, reductions in diabetes and overweight, and against malocclusion. These benefits will achieve some Sustainable Development Goals by 2030 [7]. Evidence has shown that infant gut microbiota composition and functionality are dose-dependent when breastfeeding is implemented [8].

Moreover, this early microbiota has a strong relationship with the development of adult microbiota and directly influences the metabolism and immune health [9]. Intestinal microbiota diversity and metabolic functions might result in oligosaccharide fermentation, which can lower pH levels. It has been shown that metabolites produced depend on diverse factors, such as physiological state, diet, the genotype of the organisms, and mainly the state of the gut microbiota, which is assessed by DNA sequencing given accurate data [10, 11]. Also, it will increase the production of metabolites such as short-chain fat acids, mainly acetate, butyrate, propionate, branched-chain fat acids, and other volatile organic compounds (VOCs) [12]. Interestingly, a clinical trial showed that infant diet affects metabolite composition, suggesting a relationship between the gut microbiota and the immune system [12]. It was also found that the decrease in the diversity and functionality of microbial species in the intestine has been associated with different pathologies [3]. In contrast, the infant formula contains a relatively simple oligosaccharide composition that is stable over time and does not contain milk microbiota, skin-associated taxa, or maternal antibodies [13].

Another study showed that specific bifidobacterial taxon appeared enriched in formula-fed infants, as shown by fecal metagenomics comparison between infants and their mothers [9]. This finding was explained by the differential carbon source availability in the two feeding regimes [13]. Subsequently, the gut microbiota composition and function in early life may influence immune development and association with human health and disease states [14].

This study aimed to assess how early infant breastfeeding status affects gut microbiota composition, immunoglobulin A (IgA) levels (a marker of gut immunity), and microbial production of metabolites. The study’s design compared the BF, formula feeding (FF), and combined feeding (CF).

## Materials and methods

### Registration of participants and samples

A cross-sectional study was conducted for one year. Lactating mothers were asked about their willingness to participate in this study. Written informed consent was obtained from the participants, and all the procedures followed the ethical standards of the Ethical Committee on Human Experimentation of the State Committee of Health Education and Research of the Health Secretary of San Luis Potosi, Mexico (register number SLP/007-2016). The recruitment period was extended from 1 December 2017 and finalized on 1 December 2019.

Infants were enrolled according to inclusion criteria: healthy term infants, with gestational ages of 36 to 42 weeks, ages between 1 to 6 months before the introduction of solid foods, for the CF group were infants fed with breast milk at least four times and more than twice daily. The exclusion criteria included infant antibiotic usage three months prior to being enrolled and during the study period, severe pregnancy complications, chronic metabolic and gastrointestinal diseases, major congenital anomalies, and infants with infections. Information on factors potentially influencing intestinal microbiota composition was collected with questionnaires. The infants were divided depending on their selected feeding method: BF, FF (did not include any probiotic strain), and CF. No advice or recommendations about feeding practices were expressed to the participants. Infant feces samples were collected in commercial diapers. The samples were separated into four portions and frozen at -60°C until used. Infants’ birth weight and height were obtained from hospital records. Increase in weight and height of infants till the date of enrolment over birth weight and height were calculated. Weight for height, height for age, and weight for age were calculated using WHO-Anthro Software v. 3.2.2 [15]. The mothers’ body mass index (BMI) was calculated as weight/height expressed in kg/m^2^.

### Determination of pH and fat in feces

A portion of the feces was diluted in sterile deionized water (1:10) immediately after sampling. The pH was measured using a microelectrode (Hanna Instruments, USA). The fecal fat was detected by examination under a microscope after staining with Sudan III dye.

### DNA extraction

The feces stored at -60°C were thawed and suspended in 1.5 mL suspension buffer (10 mM Tris-HCl, 1 mM EDTA, pH 8) and centrifuged at 13,000 rpm for 3 min. Then, the pellet was suspended in 200 μL of lysis buffer (10 mM Tris-HCl, 0.5% SDS, 1 mM CaCl_2_) and incubated for 30 min at room temperature with agitation. An extraction with phenol-chloroform was performed. The recovered supernatants were transferred to a new tube, one volume of chloroform was added, and the sample was centrifuged. The supernatant was precipitated with isopropanol and sodium acetate (pH 4.8) and incubated at -20°C. The obtained pellet was washed with ethanol (70%). The nucleic acids were re-suspended in 100 μL of sterile water. The extracted DNA was stored at -20°C.

### 16S rDNA Illumina MiSeq sequencing

The DNA extracted from six infant feces samples per group served as a template to prepare the 16S rDNA library following Illumina’s 16S Metagenomic Sequencing Library Preparation guide. These infants were born by vaginal delivery, and their mothers had healthy BMI in the three times mentioned above.

Briefly, PCR amplification of the V3 and V4 regions of the 16S rRNA gene (approximately 460 bp fragment) was conducted with the Phusion® High-Fidelity DNA Polymerase (New England Biolabs, Ipswich, MA, USA). Thermal cycling conditions were performed according to the manufacturer´s protocol in a Veriti 96-Well Thermal Cycler (Applied Biosystems, Foster City, CA, USA). For each biological sample, three technical PCR replicates were amplified. Positive amplification was verified by 1.5% agarose gel electrophoresis. Pooled PCR replicates were purified with Agencourt AMPure XP beads (Beckman Coulter Life Sciences, Indianapolis, IN). Library quantification and normalization were conducted with a Qubit® dsDNA HS Assay Kit (Thermo Fisher Scientific, Waltham, MA) in a Qubit 2.0 Fluorometer (Thermo Fisher Scientific, Waltham, MA). Paired-end sequencing (2x300 pb) was performed with a MiSeq Reagent Kit v3 (Illumina, San Diego, CA, USA) at a final loading concentration of 8 pM in a MiSeq System (Illumina). PhiX sequencing control (Illumina) represented 30% of the pooled library at 8 pM.

### Bioinformatic and statistical analyses

DNA sequences were analyzed using the software Mothur [16] following their Standard Operating Procedure. Initially, pair-end reads were merged into single sequences for quality filtering. Sequences were removed if they were shorter than 450 bp, with a <Q30, or possessed mismatches in the primer sequence. Sequences passing quality filters were clustered in OTUs based on a 97% sequence identity cutoff. Singletons and chimeric sequences were removed. Taxonomy was assigned by aligning OTUs to the Silva database v123.

Multivariate statistical analyses were conducted using the vegan package in R v3.3.3 (R Core Team, 2017) [17]. Read count matrixes were normalized by a square root transformation. Non-Metric Dimensional Scaling (NMDS) of phylum, family, and genus levels using Bray-Curtis dissimilarities was performed to analyze the effect of the different variables on the microbial community. Furthermore, a PerMANOVA analysis of microbial communities at the genus level was conducted using Bray-Curtis dissimilarities and 999 permutations.

### Determination of secreted IgA

The feces were homogenized with fecal dilution buffer (1:4 *w/v*) (90 mL PBS, 10 mL 0.5 M EDTA pH 8, 10 mg soybean trypsin inhibitor [Sigma-Aldrich]; 6.6 mL 100 mM PMSF [Sigma-Aldrich, dissolved in EtOH]) and shaken 30 min at 4°C. The homogenized sample was centrifuged for 15 min at 10,000 x g at 4°C, and the supernatant was collected and stored at 70°C until analysis. Before analysis, the supernatant was thawed on ice and filtrated through a 0.2 μm filter. Fecal IgA was measured in duplicate [16] using a commercial ELISA kit (Human IgA Ready-Set-Go!®, eBioscience®), following the manufacturer’s instructions.

### Analysis of VOCs

VOC content was analyzed in the fecal samples. Six infant feces samples per group were analyzed, as indicated in the samples previously used in the sequencing. Samples were analyzed in triplicate. VOCs were analyzed using a flash gas chromatography (GC) electronic nose (FGC- E-Nose) Heracles II with autosampler HS100 (AlphaMOS^®^, Toulouse, France). The Heracles II was equipped with two columns working in parallel mode: a non-polar column (DB-5: 5% phenyl- 95% dimethyl-polysiloxane and DB-1701: 14% cyanopropylphenyl- 86% dimethyl-polysiloxane). The GC injector was maintained at 200°C. The samples were subjected to a temperature program to separate the VOCs at 50°C for 30 sec, increasing at a constant speed of 10°C/ sec until 280°C. The carrier gas was hydrogen at a continuous 1 mL/min flow. Separate species were detected by electronic nose software using multivariable statistical analysis (Alpha Soft^®^ by Alpha MOS). The fecal samples were weighed with an aliquot of every single sample (approximately 200 mg) and placed in 20 mL hermetically sealed vials sealed magnetically with a plug without any treatment or extraction solvent. Samples were incubated in the autosampler for 900 sec at 40°C with constant stirring at 500 rpm, and then 1 mL of the sample was taken by the headspace in the electronic nose. A single chromatogram was created after superposing the chromatograms obtained in each column. This strategy helped reduce identification errors made by the Kovats index with the C6-C16 standard [18].

### Experimental design and statistical analyses

The data were analyzed using the Kolmogorov-Smirnov test and/or Shapiro-Wilk to determine Gaussian distribution. To determine differences among groups, we used the Kruskal-Wallis test. A post hoc analysis was performed with Dunn´s test with Bonferroni correction, and *p*-values < 0.05 were considered statistically significant. Correlation was performed with Spearman`s Rho. We analyze the data using the R software version 4.3.0 (2023-04-21), the R Foundation for Statistical Computing, and RStudio 2023.03.1 Build 446.

## Results

A total of 55 mother-infant pairs were included. The gestational age was 38.1 ± 1.8 weeks. The infants were divided into three groups: BF (n = 20), FF (n = 16), and CF (n = 19). The mother occupation mainly included housewives (BF: 60%, FF: 39%, and CF: 52%), and one mother worked in the industry (in the FF group). Regarding the place of residence, four lived in rural communities (BF:1, FF:2, and CF:1). The data of infant age at the sampling, gender, mode of delivery, gestational age, BMI of the mother (pre-gestational, pregnancy, and post-partum), were collected (S1 Table). The comparison of the groups for each parameter was evaluated; however, there were no statistically significant differences according to the one-way ANOVA test, post hoc Tuckey, or chi-square.

### Nutritional status of the infants and their mothers

Nutritional status was evaluated for each infant following the birth parameters and on the study enrolment date in weight-for-height, height-for-age, and weight-for-length. Subjects of the BF group showed the best infant development when the parameters at birth and sampling time were compared (Table 1). In contrast, the FF group tended to malnutrition and low height. Interestingly, the weight-for-age in the CF group showed a statistically significant difference compared to the FF group (*p*< 0.015, one-way ANOVA, Tukey test).

**Table 1:** Effects of feeding type on infants and the early health biomarkers.

	BF	FF	CF	*p*-value
Subject number	20	16	19	
WFL1 (median [IQR])	22.2 [8.1, 58.2]	35.6 [2.2, 52.6]	25.7 [10.6, 72.6]	0.786
WFL2 (median [IQR])	44.4 [20.6, 98.2]	22.8 [2.8, 68.0]	37.9 [24.5, 47.6]	0.255
WFA1 (median [IQR])	41.2 [25.0, 57.8]	37.9 [12.1, 64.7]	53.3 [24.9, 69.7]	0.77
WFA2 (median [IQR])	34.6 [10.7, 66.5]	9.3 [1.4, 28.9]	39.1 [33.4, 67.4]	0.015
LHFA1 (median [IQR])	64.3 [28.8, 84.7]	56.5 [43.3, 78.5]	64.3 [25.2, 81.7]	0.875
LHFA2 (median [IQR])	63.1 [10.4, 83.4]	27.0 [11.0, 66.4]	72.8 [39.2, 82.9]	0.399
pH (mean ± SD)	5.6 ±0.4	6.5 ± 0.8	6.5 ± 0.7	<0.001
Fat = n (%)	3 (15.0)	5 (31.2)	5 (26.3)	0.583

BF, breastfeeding; FF, formula feeding; CF, combined feeding; 1, sampled at birth; 2, sampled time; WFA, weight-for-age; LHFA, length/height-for-age; WFL, weight-for-length. *One-way ANOVA, post hoc Tuckey.

### Measurement of pH and fat detection in feces

The lowest pH was recorded in the BF infants, with a significant difference from the FF and CF groups (*p*< 0.001, one-way ANOVA, Tukey test). The detection of fat in feces was more common in the FF and CF groups (31% and 26%, respectively) compared to the BF group (15%); however, no statistical significance was found (Table 1).

### Measurement of IgA

The lowest levels of IgA were measured, and values of 28.7 ± 2.4, 14.0 ±1.8, and 18.9 ± 2.1 ng/mL were measured for the BF, FF, and CF groups, respectively. The data showed a parametric distribution according to Shapiro-Wilk and Kolmogorov-Smirnov normality test (*p* >0.05). The one-way ANOVA and Tukey post hoc tests found a statistically significant difference between BF vs. FF, CF, and FF vs. CF groups (*p* < 0.001). The percentile 50 were 29.0, 14.0, and 18.7 ng/mL for the BF, FF, and CF groups, respectively, with maximal values of 31.8, 16.6, and 23.3 ng/mL for BF, FF, and CF, respectively.

### Microbiota composition

**Fig 1:**
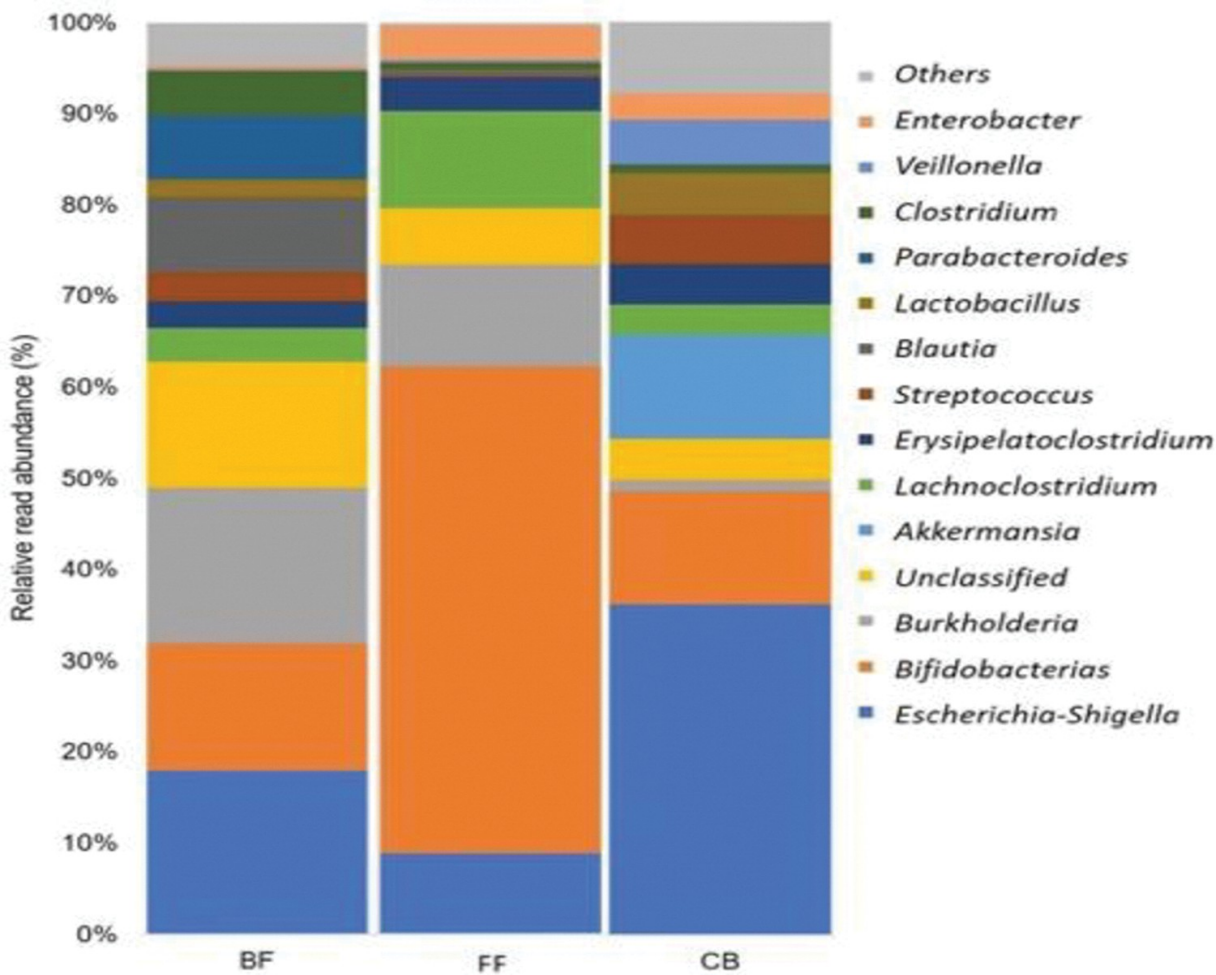
Gut microbiota analysis. Relative abundance at the genus level of the groups studied. BF, breastfeeding; FF, formula-feeding; CF, combined feeding.

The gut microbiota composition was analyzed in six representative samples of each group. Data on the percent of relative abundance of the most relevant bacterial phyla are shown, where the BF group showed more diversity in bacterial phyla (S1 Fig), with an equal distribution of around 415 species (only species occurring at > 0.1% abundance were included). At the genus level, the gut microbiota analysis showed that the genus *Blautia* was exclusively detected in the BF group. In contrast, the genus *Akkermansia* was only present in the CF group. In both BF and CF groups, the genera *Streptococcus* and *Lactobacillus* were more abundant compared to the CF group. On the other hand, the FF group presented a high abundance of the genera *Lachnoclostridium* and *Bifidobacterium*, which were also present in the other groups but with less abundance (Fig 1).

### Analysis of VOCs

**Fig 2:**
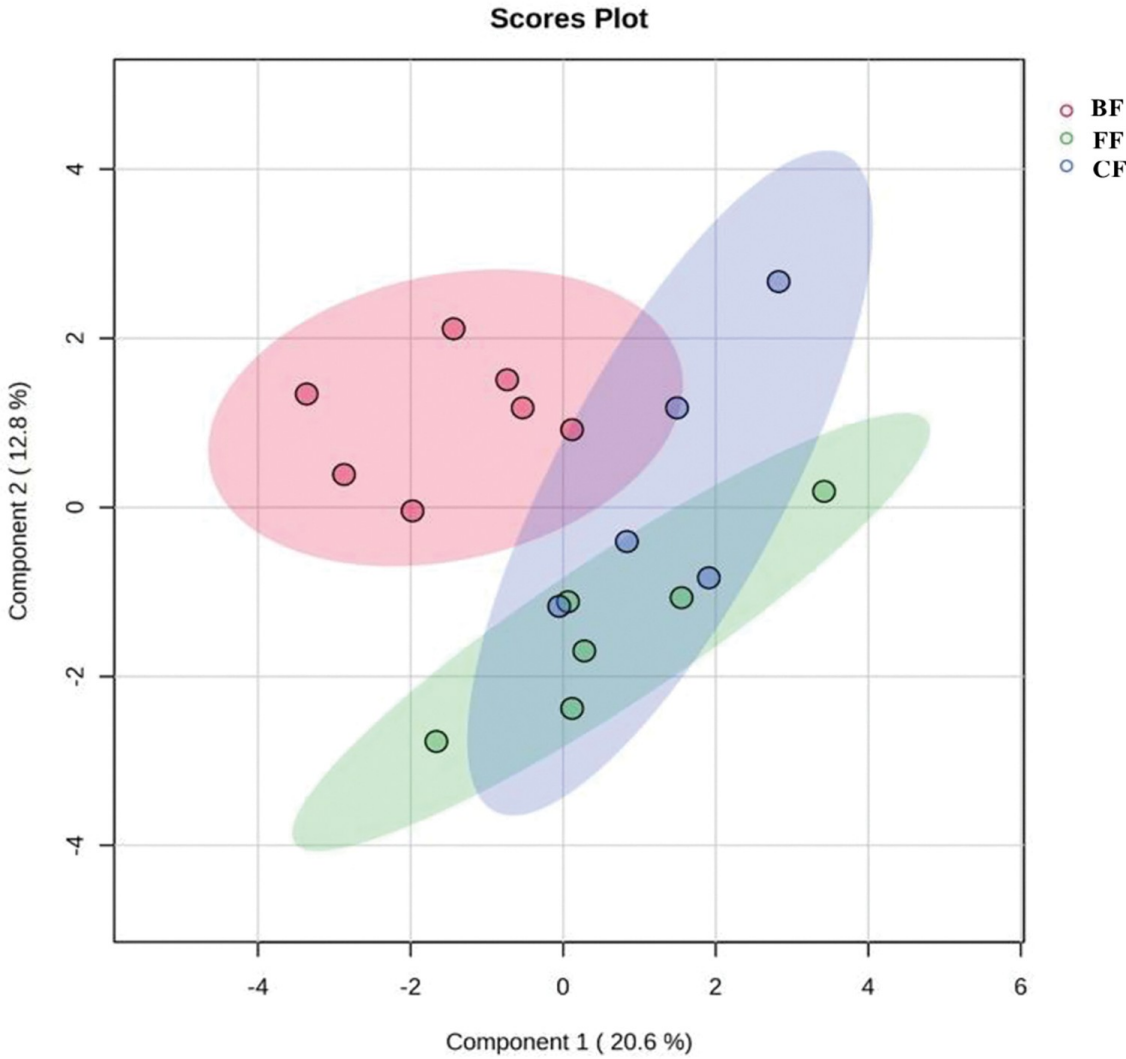
Partial Least Squares Discriminant Analysis (PLS-DA). BF, breastfeeding; FF, formula-feeding; CF, combined feeding.

**Fig 3:**
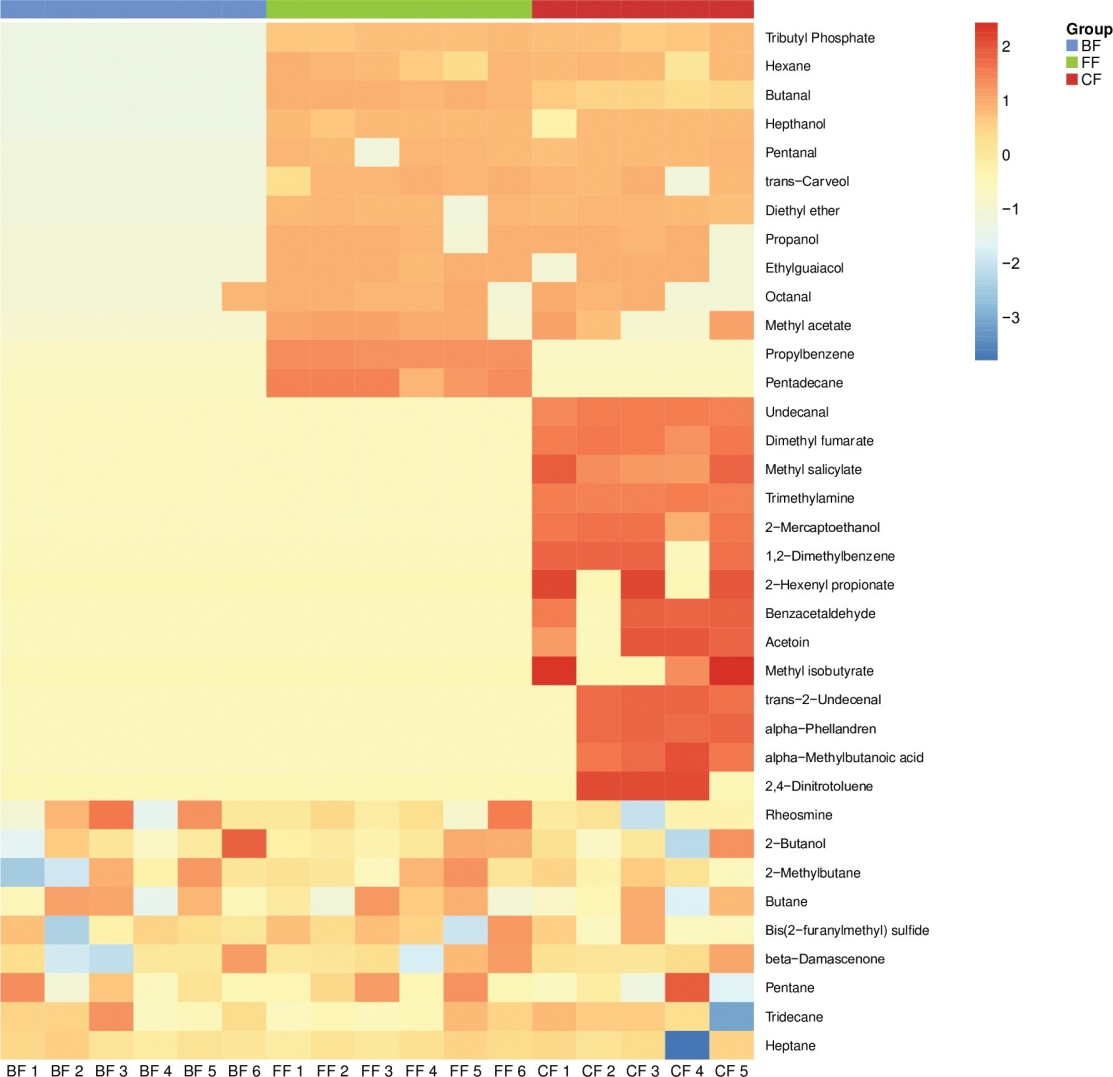
Heatmap of VOCs measured in the three groups studied. BF, breastfeeding; FF, formula-feeding; CF, combined feeding.

GC electronic nose (FGC- E-Nose) was utilized to analyze metabolites in six infant feces samples per group. The resulting chromatograms detected 36 VOC metabolites (Table 2), comparison between the three groups of feeding showed a statistical difference of *p*-value <0.001when Butanal was compared between BF vs. FF, as well as the separation trend for each group was detected from a Partial Least Squares Discriminant Analysis (PLS-DA) (Fig 2). A heat map was plotted (Fig 3).

**Table 2:** Comparison between the 3 groups of feeding.

VOC	*p*-value	Post hoc significance		
		BF *vs*. CF	BF *vs*. FF	CF *vs*. FF
1,2-Dimethylbenzene	0.003	**	ns	**
2-Butanol	0.813	ns	ns	ns
2-Hexenyl propionate	0.0171	*	ns	*
2-Mercaptoethanol	< 0.001	**	ns	**
2-Methylbutane	0.644	ns	ns	ns
2,4-Dinitrotoluene	0.017	*	ns	*
3-Methylbutanoic acid	<0.001	**	ns	**
Acetoin	0.003	**	ns	**
α-Phellandren	0.003	**	ns	**
Benzacetaldehyde	0.0031	**	ns	**
β-Damascenone	0.413	ns	ns	ns
Bis(2-furanylmethyl) sulfide	0.367	ns	ns	ns
Butanal	<0.001	ns	***	ns
Butane	0.913	ns	ns	ns
Diethyl ether	0.008	*	*	ns
Dimethyl fumarate	<0.001	**	ns	**
Ethyl guaiacol	0.004	ns	**	ns
Heptane	0.95	ns	ns	ns
Hepthanol	0.003	*	*	ns
Hexane	0.003	*	**	ns
Methyl acetate	0.027	ns	*	ns
Methyl isobutyrate	0.017	*	ns	*
Methyl salicylate	<0.001	**	ns	**
Octanal	0.104	ns	ns	ns
Pentadecane	<0.001	ns	**	**
Pentanal	0.007	*	*	ns
Pentane	0.049	ns	ns	ns
Propanol	0.016	ns	*	ns
Propylbenzene	<0.001	ns	**	**
Rheosmine	0.334	ns	ns	ns
*trans*-2-Undecenal	<0.001	**	ns	**
*trans*-Carveol	0.006	ns	**	ns
Tributyl phosphate	0.003	*	**	ns
Tridecane	0.737	ns	ns	ns
Trimethylamine	<0.001	**	ns	**
Undecanal	<0.001	**	ns	**

*, *p*-value<0.05

**: *p*-value < 0.01

*** *p*-value <0.001. ns, no significant.

**Fig 4:**
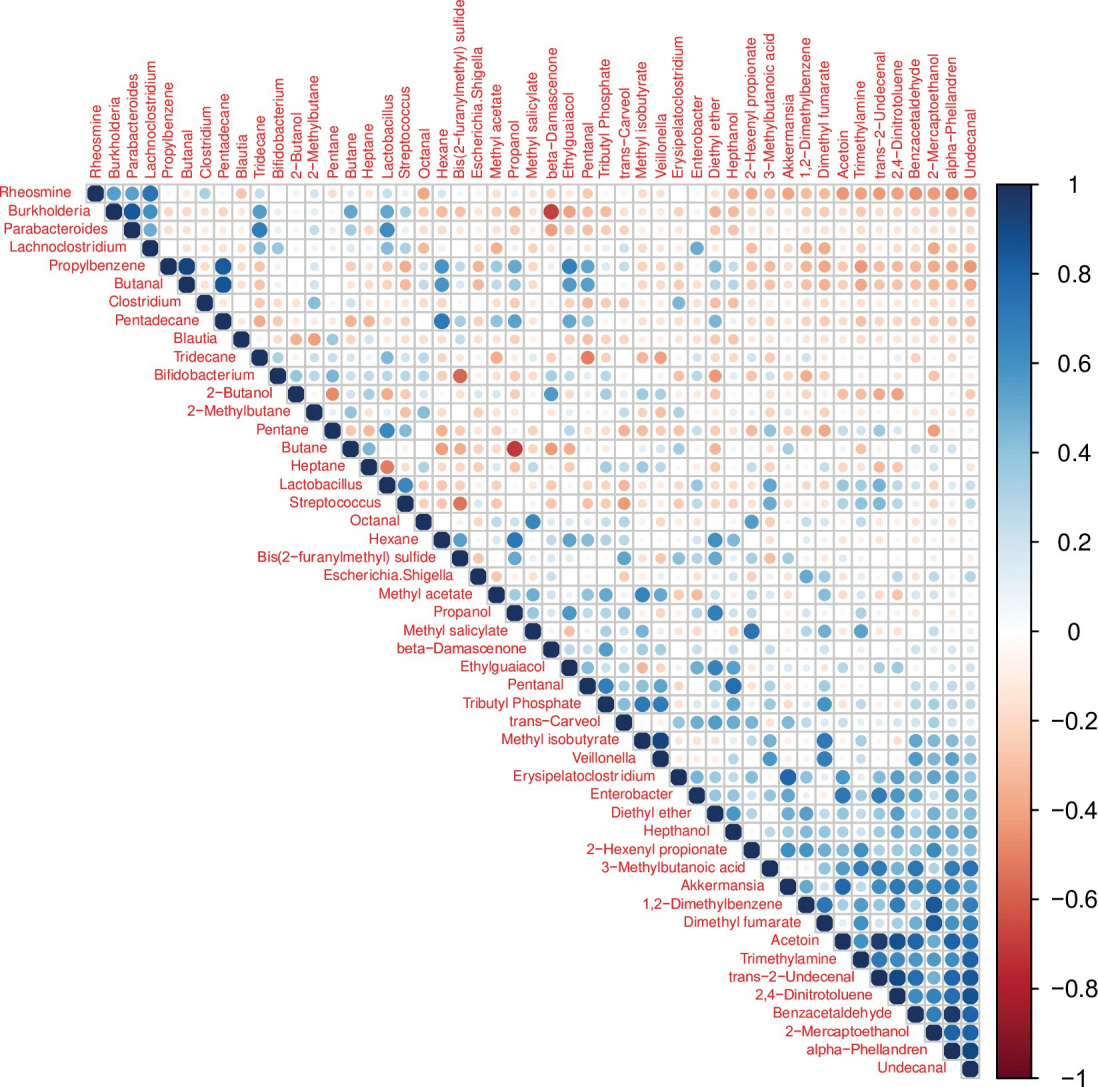
Heatmap showing the correlation of VOCs detected with the main bacterial genera from infants in feces samples. Circles represent correlation intensity performed with Spearman’s Rho. Blue circles indicate a positive relation, whereas red circles indicate a negative relation.

We examined the correlation of the VOCs detected in the feces samples with the main bacterial genera from infant gut microbiota (Fig 4).

The genera *Lachnoclostridium* and *Parabacteroides* were positively correlated to rheosmine and tridecane. In addition, *Bifidobacterium* and *Streptococcus* were negatively correlated to bis(2-furanylmethyl) sulfide, whereas *Burkholderia* was negatively correlated to β-damascenone. Interestingly, the genus *Blautia* was negatively associated with 2-methylbutane and 2-butanol. Moreover, the genus *Akkermansia* found only in the CF group, was also negatively associated with rheosmine.

From the list of the 36 VOCs detected in the study, our analysis was further focused on health biomarkers of intestinal diseases. These VOCs included 3-methylbutanoic acid, butanal, pentadecane, propylbenzene, *trans*-2-undecenal, and trimethylamine (Table 3).

**Table 3:** Comparison of selected VOCs between the 3BF, FF, and CF groups.

VOC	*p*-value	Post hoc significance			Source		Associated disorders and diseases related to the gut	Type of sample
		BF *vs* CF	BF *vs* FF	CF *vs* FF				
3-Methylbutanoic acid	<0.001	**	ns	**	M	-	Celiac disease	Feces, urine [19]
Butanal	<0.001	ns	***	ns	M	-	Ulcerative colitis	Feces [20]
							Crohn’s disease	
Pentadecane	<0.001	ns	**	**	-	E	Celiac disease	Saliva [21]
Propylbenzene	<0.001	ns	**	**	M	-	*Campylobacter jejuni* infection	Feces [20]
*trans*-2-Undecenal	<0.001	**	ns	**	-	E	Asthma	Breath [22]
Trimethylamine	<0.001	**	ns	**	M	E	Trimethylaminuria	Urine [23]
							Crohn’s disease	Fecal [24]
							Ulcerative colitis	Fecal [24]

*, *p*-value <0.05

**, *p*-value <0.01

*** *p*-value <0.001. BF, breastfeeding; FF, formula-feeding; CF, combined feeding. M, microbiota; E, exogenous; ns, no significant.

An analysis post hoc was carried out to assess the significance between the groups. The compounds 3-methylbutanoic acid, *trans*-2-undecenal, and trimethylamine showed no significance when the BF and FF were compared; however, the Human Metabolome Database reported that those compounds have a relationship to disorders and gut diseases (Table 3) [25]. The compound butanal showed a high statistical difference when comparing the BF and FF. Another study showed that this compound was found in the feces of patients with *Campylobacter jejuni* infections, where butanal was reported in 95% of the cases [20]. Moreover, another study reported the identification of butanal in 11 of 83 patients with Crohn´s disease [26].

The compounds pentadecane [21] and propylbenzene [20], reported as related to gut disorders and diseases, showed no statistical difference when BF and CF groups were compared. However, a significant difference was found when compared to the FF group.

## Discussion

Early gut colonization has been shown to have long-lasting consequences. These consequences are related to an increased risk of developing malnutrition and metabolic or immunological disease in late life [27]. The intestinal microbiota is known to influence the development and balance of the host immune system. It has been implicated in preventing damage induced by opportunistic microbes, repairing damage to the mucosal barrier, and influencing systemic autoimmune diseases [28]. The mode of delivery has a direct influence on the infant’s intestinal microbiota, an important concern is the increase in the number of deliveries by cesarean section, as well the administration of prophylactic antibiotics, which could be related to an increased risk of allergic illness [29]. In our study 52% of the infants were born by cesarean section, however, all of them were in accordance with the exclusion criteria about the infant antibiotic usage three months prior to be enrolled and during the study period. The specific microorganisms’ relevance and impact on health have been presented with the typical probiotic microorganisms and segmented filamentous bacteria of the phylum Firmicutes, associated with higher levels of Th 17 cells [30]. Although the most abundant human milk proteins are casein, lactoferrin, secreted IgA, lysozyme, and serum albumin, the protein level decreases in human milk over the 4 to 6 weeks of life [28]. The groups in our study had an average age of 3 months, whereby we assume that the concentration of IgA in the feces is in the range that represents the highest level of secreted IgA in the gut. In this regard, a case-control study that included full-term newborns with biopsy-verified and diagnosed celiac disease (CD) and healthy infants showed that microbiota establishment developed with increased bacteria diversity in healthy control compared to infants who developed CD [31]. Our results are aligned with this study because the BF group showed the highest level of secreted IgA.

The differential carbon source availability has been postulated as a factor in the bacterial diversity in the BF and FF [13]. This assumption is based on studies about the difference between HMO in BF and FF’s non-digestible carbohydrates (NDCs). The beneficial effects of HMO could be in both gut microbiota and direct effects on the immune system [11]. However, more studies are necessary to clarify the direct effect of the immune cells and its benefits related to NDCs, which have been reported to give a transition from T Helper 2 to T Helper 1 cells. It has been hypothesized that the disturbance of this transition could be related to proinflammatory cytokine production, which increases the risk of development disorders and diseases in later life [32]. On the other hand, the difference in the percent of relative abundance of phyla between the three groups studied agrees with a previous report [13].

In this study, the microbiota of the infants in each group and the VOCs detected differed. The microbiota diversity in samples of the BF group was higher than in the groups FF and CF. Specific differences between the groups were detected when the gut microbiota was analyzed at the phylum and genera levels. The BF group showed a high abundance in the phylum Firmicutes, which includes *Lactobacillus*, *Clostridium*, *Streptococcus*, *Staphylococcus*, and *Blautia*. Moreover, the genus *Blautia* was detected exclusively in the BF group, which has been postulated as a new probiotic strain for its regulatory role in diseases [33]. Moreover, *Lactobacillus* and *Streptococcus* were found more abundant in the CF group, and the genus *Akkermansia* was detected exclusively in this group. Interestingly, this genus is associated with human health since it can degrade to HMO to promote syntrophy with beneficial bacteria, contributing to developing a microbial network in the gut [34].

VOCs are ubiquitous in our environment [35] with exposure and accumulation to breastfeeding mothers in daily life [36]. These chemicals are present in breast milk and infant formula, which infants consume [37], influencing fecal VOC composition [38]. This effect can be exacerbated when mothers spend longer in outdoor activities, workplaces, or smoking habits [38]. Other sources of VOCs are the exposure to hair dyes [39], hair and skin personal care products, etc. [39].

Although it is well known that many health benefits have been demonstrated to be associated with human milk [40], such as developing neuronal and intestinal cells and strengthening the immune system in infants [41]. This assumption is based on breastfeeding because this group showed a decrease or absence of these compounds. Thus, the BF group can offer protection against the toxicity of VOCs, which can be exacerbated if the infants are less exposed to polluted environments [42].

The CF group showed a reduced level of specific VOCs, such as pentadecane [21] and propylbenzene [19], compared with the FF group, which were related to disorders and diseases of the gut. The findings of our study highlight the need for larger studies to provide insights into the exposure of the infant population since these compounds may alter the human microbiota [43], and the exposure to these compounds should be limited during the early first months of life [44].

**Fig 5:**
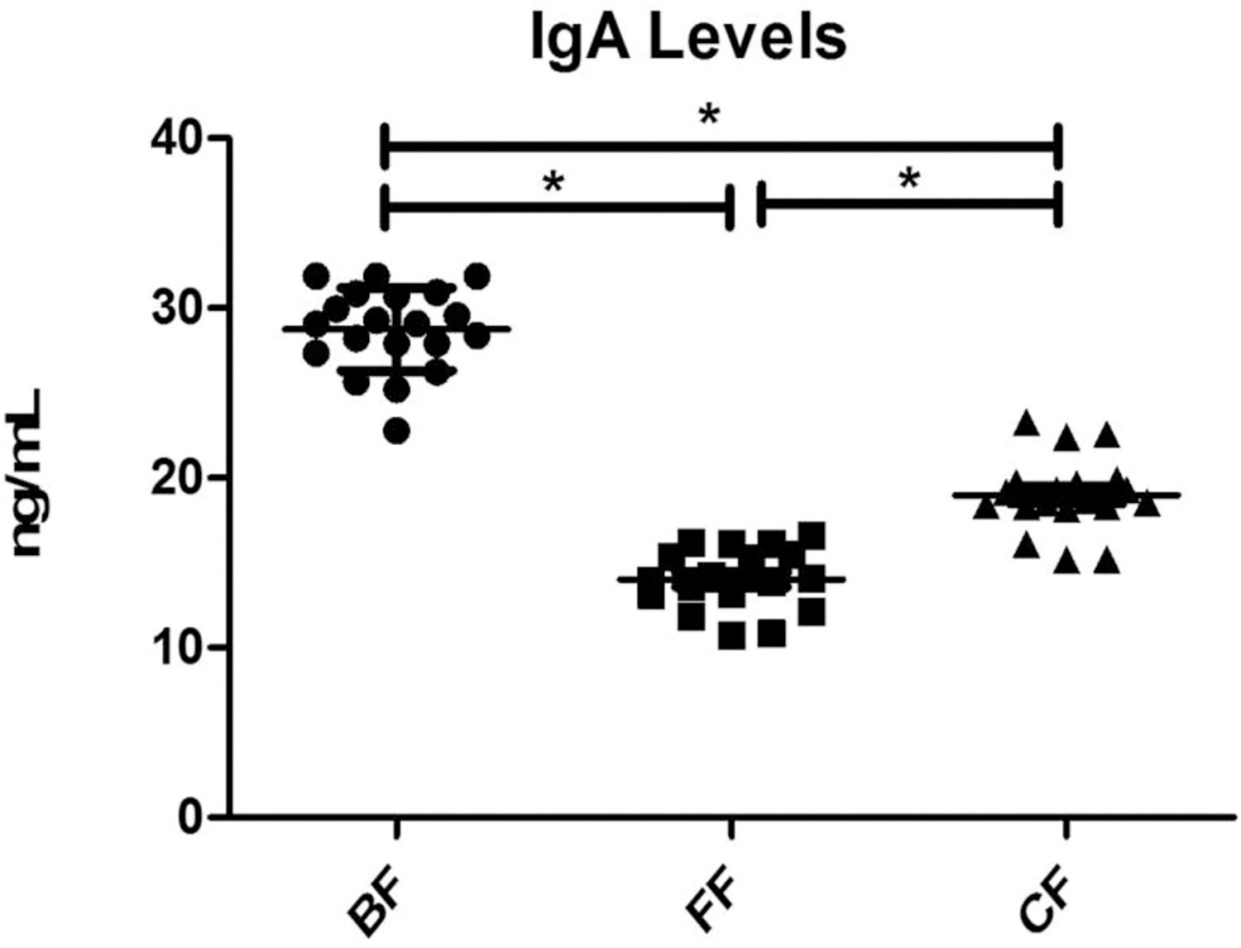
Level of IgA in the different groups. The level of IgA was measured in the feces of the three modalities tested in this study. The one-way ANOVA and Tukey post hoc tests found a statistically significant difference between BF to FF, CF, and FF to CF groups (*p* < 0.001). BF, breastfeeding; FF, formula-feeding; CF, combined feeding.

Our findings also showed that the BF group at an early age is associated with an increase in fecal IgA levels. This increase can be explained by the colostrum provided to the infant by the mother at birth. Colostrum is a fluid rich in maternal antibodies that protect the newborn from infection and boost the immune system upon BB in the first life months. In addition, milk provides immune-developmental effects in infants via direct changes in their intestinal mucosal responses. In addition, the measurement of a higher level of IgA in the BF group suggests a modulation of the microbiota, including its composition and development (Fig 5). However, children without BF can produce IgA by themselves, but at much lower levels.

## Conclusion

Our study reveals benefic effects such as microbiota diversity, IgA levels, moreover, the child´s growth in the BF group showed the best infant development when the data were compared at birth to the recollection time, as noted by the correlation with a decreased concentration of toxic volatile organic compounds. Interestingly, the CF group showed a significant difference in health status when the data were compared with the FF group. We conclude that early health practices influence children’s growth, which is relevant to further research about how those infants’ health evolved. Although the size of the sample limits this study, it provides evidence of new valuable observations, large prospective cohort studies would provide evidence of the evolution of the health of those infants and the impact of the early intervention, not only through the diet, in the next stages of growth.

## Supporting information

S1 Fig Diversity in bacterial phyla and relative abundance in the feeding modalities. (PPTX)

S1 Table General information of the participants before starting the study (mean ± SD). (DOCX)
