# Supplementary figures and images for: Influence of feeding practices in the composition and functionality of infant gut microbiota and its relationship with health
Source: PLoS One. 2024 Jan 3;19(1):e0294494. doi: 10.1371/journal.pone.0294494 (PMC10763948; doi:10.1371/journal.pone.0294494)

## Slide 1
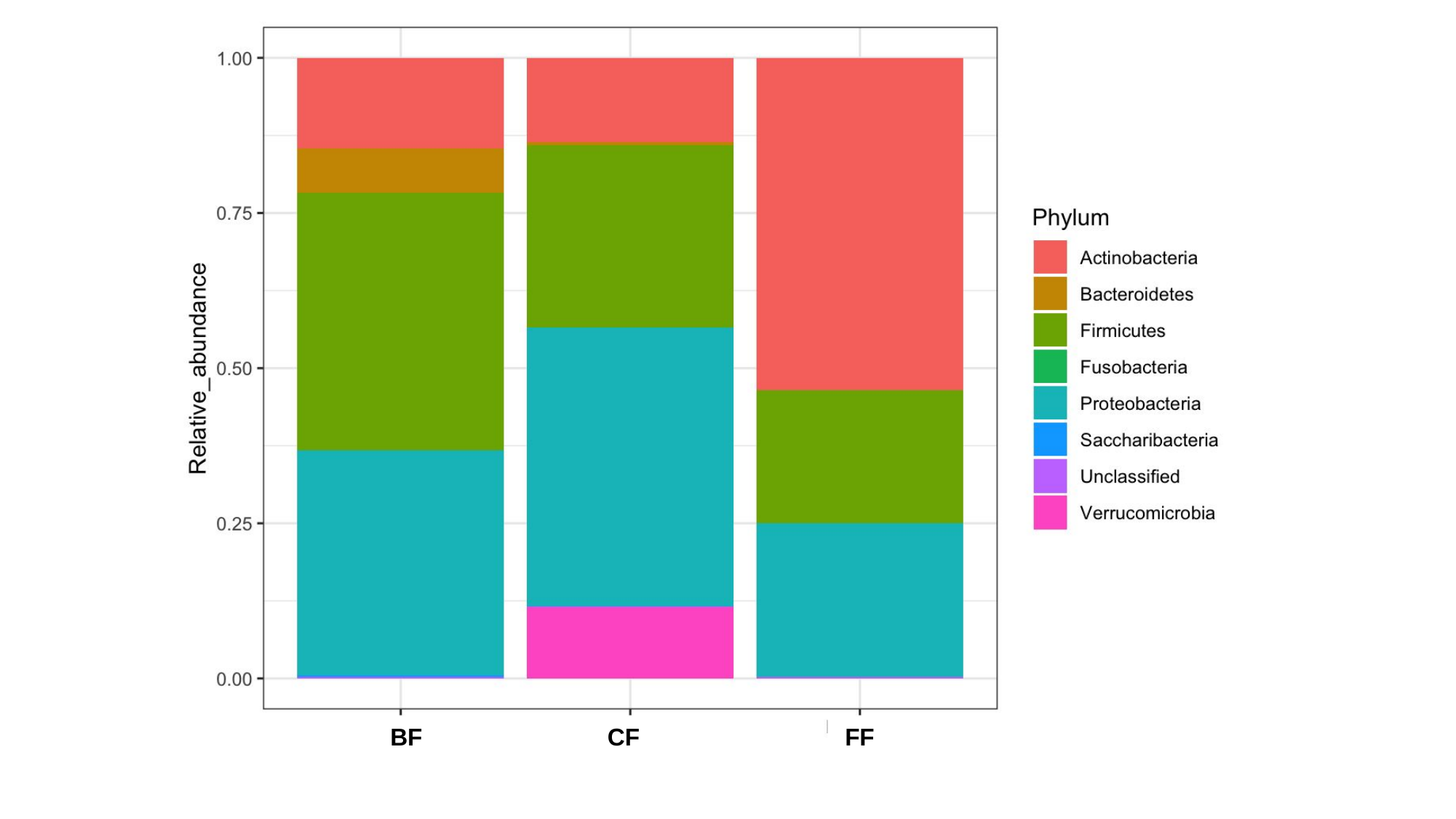

CF
 FF
BF

Supplement: S1 Fig — (PPTX) [file pone.0294494.s001.pptx]
