# Supplementary material for: Influence of feeding practices in the composition and functionality of infant gut microbiota and its relationship with health
Source: PLoS One. 2024 Jan 3;19(1):e0294494. doi: 10.1371/journal.pone.0294494 (PMC10763948; doi:10.1371/journal.pone.0294494)
Supplement: S1 Table — (DOCX) [file pone.0294494.s002.docx]

| **Group** | **Age**  **(months)** | **Gender (females)** | **Delivery mode**  **(cesarean)** | **Gestational**  **time (weeks)** | **BMI of mother**  **(kg/m^2^)** |
| --- | --- | --- | --- | --- | --- |
|  |  |  |  |  | **PG P PP** |
| **BF** | 3.3±1.7 | 20 (8) | 20 (11) | 38.4±2.1 | 23.6±2.5 28.3±2.8 24.3 ±2.9 |
| **FF** | 3.4±1.8 | 16 (8) | 16 (8) | 37.7±1.6 | 24.0±3.8 29.3±4.0 26.4 ±3.8 |
| **CF** | 3.3±1.9 | 19 (11) | 19 (10) | 38.2±1.8 | 25.27±4.4 28.31±4.3 25.1 ±4.7 |
| **p** | 0.9298 ^a^ |  | 0.6314 ^b^ |  | 0.425^a^ 0.4993^a^ 0.1762^a^ |

**S1 Table. General information of the participants before starting the study (mean ± SD).**

BF, breastfeeding; FF, formula feeding; CF, combined feeding; PG, pre-gestational; P, pregnancy; PP, postpartum; BMI, Body Mass Index. *Date of the last dose at least 7 days before sampling. ^a^ One-way ANOVA, post hoc Tuckey. ^b^ chi-square.
